# Impact of in vivo T cell depletion in HLA-identical allogeneic stem cell transplantation for acute myeloid leukemia in first complete remission conditioned with a fludarabine iv-busulfan myeloablative regimen: a report from the EBMT Acute Leukemia Working Party

**DOI:** 10.1186/s13045-016-0389-4

**Published:** 2017-01-24

**Authors:** Marie Thérèse Rubio, Maud D’Aveni-Piney, Myriam Labopin, Rose-Marie Hamladji, Miguel A. Sanz, Didier Blaise, Hakan Ozdogu, Etienne Daguindeau, Carlos Richard, Stella Santarone, Giuseppe Irrera, Ibrahim Yakoub-Agha, Moshe Yeshurun, Jose L. Diez-Martin, Mohamad Mohty, Bipin N Savani, Arnon Nagler

**Affiliations:** 1 Service d’Hématologie et de Médecine interne, Hôpital Brabois, CHRU Nancy, Nancy, France; 2 IMoPA, CNRS UMR 7365, Nancy, France; 3 Université de Lorraine, Nancy, France; 4 ALWP Office, Hôpital Saint Antoine, Paris, France; 5 Service d’Hématologie et de Thérapie Cellulaire, Hôpital Saint Antoine, Paris, France; 6 INSERM UMR 938, Paris, France; 7 Université Pierre et Marie Curie, Paris, France; 8 Service Hématologie Greffe de Moëlle, Centre Pierre et Marie Curie, Alger, Algeria; 9 Servicio de Hematologia, Hospital Universitario La Fe, Valencia, Spain; 10 Programme de Transplantation and Therapie Cellulaire, Centre de Recherche en Cancérologie de Marseille, Institut Paoli Calmettes, Marseille, France; 11 Hematology Division, BMT Unit, Hematology Reserach Laboratory, Training and Medical, Baskent University Hospital, Adana, Turkey; 12 Hopital Jean Minjoz, Service d`Hématologie, Besancon, France; 13 Servicio de Hematología-Hemoterapia, Hospital U. Marqués de Valdecilla, Santander, Spain; 14 Dipartimento di Ematologia, Medicina Trasfusionale e Biotecnologie, Ospedale Civile, Pescara, Italy; 15 Azienda Ospedaliera, Centro Unico Regionale Trapianti, Reggio, Calabria Italy; 16 Hôpital HURIEZ UAM allo-CSH, CHRU, Lille, France; 17 Hematology and BMT Department, Beilinson Hospital, Petach-Tikva, Israel; 18 Sección de Transplante de Medula Osea, Hospital Gregorio Marañón, Madrid, Spain; 19 Vanderbilt University Medical Center, Nashville, TN USA; 20 Division of Hematology, Chaim Sheba Medical Center, Tel Hashomer, Israel

**Keywords:** Allogeneic stem cell transplantation, HLA-matched related donor, Acute myeloid leukemia, In vivo T cell depletion, Graft-versus-host disease, Relapse incidence, GRFS

## Abstract

**Background:**

The impact of the use of anti-thymocyte globulin (ATG) in allogeneic stem cell transplantation performed with HLA-identical sibling donors following fludarabine and 4 days intravenous busulfan myeloablative conditioning regimen has been poorly explored.

**Methods:**

We retrospectively analyzed 566 patients who underwent a first HLA-identical allogeneic stem cell transplantation with this conditioning regimen for acute myeloid leukemia in first complete remission between 2006 and 2013 and compared the outcomes of 145 (25.6%) patients who received ATG (ATG group) to 421 (74.4%) who did not (no-ATG group). The Kaplan-Meier estimator, the cumulative incidence function, and Cox proportional hazards regression models were used where appropriate.

**Results:**

Patients in the ATG group were older, received more frequently peripheral blood stem cell grafts from older donors, and were transplanted more recently. With a median follow-up of 19 months, patients in the ATG group had reduced 2-year cumulative incidence of chronic graft-versus-host disease (GVHD) (31 vs. 52%, *p* = 0.0002) and of its extensive form (8 vs. 26%, *p* < 0.0001) but similar relapse incidence (22 vs. 27%, *p* = 0.23) leading to improved GVHD and relapse-free survival (GRFS) (60 vs. 40%, *p* = 0.0001). In multivariate analyses, the addition of ATG was independently associated with lower chronic GVHD (HR = 0.46, *p* = 0.0001), improved leukemia-free survival (HR = 0.67, *p* = 0.027), overall survival (HR = 0.65, *p* = 0.027), and GRFS (HR = 0.51, *p* = 4 × 10^−5^). Recipient age above 50 years was the only other factor associated with worse survivals.

**Conclusions:**

These results suggest that the use of ATG with fludarabine and 4 days intravenous busulfan followed by HLA-identical sibling donor allogeneic stem cell transplantation for acute myeloid leukemia improves overall transplant outcomes due to reduced incidence of chronic GVHD without increased relapse risk.

**Electronic supplementary material:**

The online version of this article (doi:10.1186/s13045-016-0389-4) contains supplementary material, which is available to authorized users.

## Background

Allogeneic hematopoietic stem cell transplantation (allo-SCT) with myeloablative conditioning (MAC) regimen remains the treatment of choice for intermediate or poor-risk acute myeloid leukemia (AML) in first complete remission (CR1) [1]. The standard conditioning regimen including myeloablative dose of intravenous (iv) busulfan and cyclophosphamide is however associated with substantial toxicities in adults above 40 years of age [2]. The association of fludarabine to myeloablative dose of iv busulfan (Flu-ivBu4), developed in the past two decades, has been shown to preserve significant anti-leukemic activity with reduced toxicity mortality in both retrospective [3–8] and prospective randomized studies [9, 10], in particular, in adults above 40 years. This so-called reduced toxicity conditioning (RTC) regimen is therefore being widely used in allo-SCT for patients with AML in CR1.

The Flu-ivBu 4 regimen is usually performed with peripheral blood stem cells (PBSC) to favor engraftment and enhance the graft-versus-leukemia (GVL) effect [11, 12]. However, use of PBSC from HLA-matched related (MRD) or unrelated (MUD) donors with MAC increases the risk of chronic graft-versus-host disease (cGVHD) [11–13]. Prospective randomized studies have shown that in vivo T cell depletion with anti-thymocyte globulin (ATG) reduces the incidence of cGVHD without increasing the risk of relapse in allo-SCT performed with PBSC from MRD or MUD after conventional cyclophosphamide-based MAC regimens for AML [14–16]. These results raise the question of the impact of use of ATG in the Flu-ivBu4 RTC, in which the balance between the GVH and GVL effects of allo-SCT might be more sensitive to T cell depletion. Very scarce data exist on the potential effect of ATG in this transplant context. Russel et al. reported reduced non-relapse mortality (NRM) due to lower incidence of cGVHD in a retrospective study of patients transplanted with MRD after Flu-Bu-based MAC and ATG compared to conventional cyclophosphamide-based MAC without ATG but a trend towards higher relapse incidence [17]. A Korean study comparing the outcomes of 16 patients receiving Flu-ivBu for 3 or 4 days and ATG to 45 patients receiving the same type of conditioning without ATG for various hematological malignancies transplanted with MRD did not observe any benefit of adding ATG, with also concerns about its possible negative impact on relapse [18]. Heterogeneity in terms of conditioning between groups or of types of disease limits the interpretation of these data. With the objective to explore the impact of the use of ATG in the Flu-ivBu4 RTC, we chose to retrospectively analyze a cohort of 566 adult patients given hematopoietic stem cells from HLA-identical sibling donors for AML in CR1 following Flu-ivBu4 conditioning regimen. In this homogeneous cohort of patients, we compared post-transplant outcomes of 145 of those who received ATG for GVHD prophylaxis to the 421 patients who did not.

## Methods

### Study design and data collection

This is a retrospective multicenter analysis using the data set of the Acute Leukemia Working Party (ALWP) of the European Society of Blood and Marrow Transplantation (EBMT) group registry. The EBMT is a voluntary working group of more than 500 transplant centers that are required to report all consecutive stem cell transplantations and follow-ups once a year. Audits are routinely performed to determine the accuracy of the data. The study was planned and approved by the ALWP of the EBMT. In addition, the study protocol was approved by the institutional review board at each site and complied with country-specific regulatory requirements. The study was conducted in accordance with the Declaration of Helsinki and Good Clinical Practice guidelines. Since 1990, patients provide informed consent authorizing the use of their personal information for research purposes. Eligibility criteria for this analysis included adult patients above 18 years of age with AML who underwent a first allo-SCT from an HLA-matched related donor following fludarabine and 4 days of intravenous busulfan (Flu-ivBu4) regimen between 2006 and 2013. Exclusion criteria were previous allogeneic or cord blood transplantation and ex vivo T cell-depleted stem cell graft. Variables collected included recipient and donor characteristics (age, gender, CMV serostatus), disease characteristics and status at transplant, year of transplantation and interval from diagnosis to transplantation, transplant-related factors including conditioning regimen, use and dose of thymoglobulin as pre-transplant in vivo T cell depletion, stem cell source (bone marrow (BM) or peripheral blood (PB)), post-transplant GVHD prophylaxis. GVHD prophylaxis regimens were dependent on centers’ protocols. Grading of acute GVHD was performed using established criteria [19]. Chronic GVHD was classified as limited or extensive according to published criteria [20]. For the purpose of this study, all necessary data were collected according to the EBMT guidelines, using the EBMT minimum essential data forms. The list of institutions reporting data included in this study is provided in the supplemental data (Additional file 1: Table S1).

### Statistical analysis

Study end points were engraftment, incidences and severity of acute and chronic GVHD, incidence of primary disease relapse (RI), NRM, leukemia-free survival (LFS), overall survival (OS), and GVHD and relapse-free survival (GRFS). Start time was date of transplant for all end points. LFS was defined as survival without relapse or progression, NRM as death without relapse/progression, and GRFS as survival with no evidence of relapse/progression, grade III to IV acute graft-versus-host disease (aGVHD), or severe cGVHD as defined by Ruggeri et al. for registry-based studies [21]. Cumulative incidence functions (CIF) were used to estimate RI and NRM in a competing risk setting, because death and relapse compete with each other. For estimating the cumulative incidence of chronic GVHD, we considered relapse and death to be competing events. Groups were compared by the chi-square method for qualitative variables, whereas the Mann-Whitney test was applied for continuous parameters. Univariate comparisons were done using the log-rank test for OS, LFS, and GRFS and the Gray’s test for RI, NRM, and GVHD cumulative incidences. Multivariate analyses were performed using Cox proportional hazards model for all end points. Factors differing in terms of distribution between the groups and all factors known as potentially risk factors were included in the final model. In order to test for a center effect, we introduced a random effect or frailty for each center into the model [22]. All tests were two-sided. The type I error rate was fixed at 0.05 for the determination of factors associated with time to event outcomes. Statistical analyses were performed with SPSS 22.0 (IBM Corp., Armonk, NY, USA) and R 3.2.3 software packages (R Development Core Team, Vienna, Austria).

## Results

### Patient, transplant, and disease characteristics

Between 2006 and 2013, 566 patients with AML transplanted with a sibling donor following a Flu-ivBu4 myeloablative conditioning regimen with or without ATG were included in the study. Patient and disease characteristics are summarized in Table 1. Among the total population of patients, 421 (74.4%) did not receive ATG within the conditioning regimen (no-ATG group), while 145 (25.6%) received ATG (ATG group). Thymoglobulin was the main ATG brand used (95.2%). Median dose of thymoglobulin was 5 mg/kg (range, 2.5–15.8), and a majority of patients (73.7%) received a total dose below 6 mg/kg. Apart from ATG, GVHD prophylaxis was mainly based on the association of cyclosporine (CsA) and methotrexate (MTX) in the no-ATG group (88.4%), while most of the patients in the ATG group received CsA alone (29%) or CsA and MTX (40%). The choice of GVHD prophylaxis was dependent on the centers’ protocols.

**Table 1:** Patient and disease characteristics

Patient characteristics	No-ATG	ATG	*p* value
Number of patients	421	145	
Recipient age at SCT (years, range)	43.7 (18–68)	48.8 (20–69)	0.002
Recipient gender, *n* (%)			0.24
Male	215 (51.3%)	82 (56.9%)	
Female	204 (48.7%)	62 (43.1%)	
Year of SCT (median), year (%)	2011 (2006–2013)	2012 (2006–2013)	<10^−5^
Interval from diagnosis to SCT (median days)	156	156	0.79
Median follow-up ^a^ (months, range)	16 (1.5–93)	21 (1–106)	0.81
Donor age (years, range)	41 (8–70)	47 (10–65)	0.003
Donor gender, *n* (%)			
Male	236 (56.3%)	70 (48.6%)	0.11
Female	183 (43.7%)	74 (51.4%)	
Female donor to male recipient, *n* (%)	96 (23%)	44 (30.8%)	0.06
Diagnosis, *n* (%)			0.05
De novo AML	391 (92.9%)	127 (87.6%)	
Secondary AML	30 (7.1%)	18 (12.4%)	
Cytogenetics in de novo AML, *n* (% of available data)			0.60
Good	18 (16.2%)	6 (9%)	
Intermediate	76 (68.5%)	50 (74.6%)	
Poor	17 (15.3%)	11 (16.4%)	
Not available/failed	280	60	
Source of SC, *n* (%)			<10^−4^
BM	84 (20%)	10 (6.9%)	
PB	337 (80%)	135 (93.1%)	
In vivo T cell depletion, *n* (%)			
Thymoglobuline	0	138 (95.2%)	
ATG Fresenius		4 (2.8%)	
Missing brand of ATG		3 (2%)	
Mean dose of thymoglobuline (mg/kg) (range)		5 (2.5–15.8)	
Thymo ≤ 6 mg/kg		98 (73.7%)	
Thymo > 6 mg/kg		35 (26.3%)	
Unknown dose of thymoglobuline		12	
Post-transplant GVHD prophylaxis			<10^−4^
CsA	4 (1%)	42 (29%)	
CsA + MTX	372 (88.4%)	57 (39.3%)	
CsA/FK 506 + MMF	35 (8.3%)	17 (11.7%)	
Other	10 (2.4%)	29 (20%)	
Patient positive CMV serology, *n* (%)	362 (87%)	107 (74.3%)	<10^−4^
Donor positive CMV serology, *n* (%)	330 (80.1%)	104 (72.7%)	0.07
CMV risk, *n* (%)			0.008
Low	33 (8%)	22 (15.5%)	
Intermediate	328 (77.9%)	103 (71%)	
High	49 (12%)	17 (12%)	

CMV risk low = negative recipient and donor serology, high positive recipient and negative donor serology, intermediate: all other combinations

*AML* acute myeloid leukemia, *ATG* anti-thymocyte globulin, *BM* bone marrow, *CMV* cytomegalovirus, *CsA* cyclosporine A, *MMF* mycophenolate mofetil, *MTX* methotrexate, *PB* peripheral blood, *SC* stem cells, *SCT* stem cell transplantation

^a^For alive patients

In comparison to the no-ATG group, patients in the ATG group were older (median age of 48.8 vs. 43.7 years, *p* = 0.002), had been transplanted more recently (median year of transplantation 2012 vs. 2011, *p* < 10^−5^), with older donors (median age of 47 vs. 41 years, *p* = 0.003), and were more frequently transplanted with PBSC graft (93 vs. 80%, *p* < 10^−4^) for secondary AML (12 vs. 7%, *p* = 0.05). There was no difference in terms of cytogenetic risk between the two groups in patients with available cytogenetics. Significantly higher proportions of CMV seropositive patients were transplanted in the no-ATG group (87 vs. 74%, *p* < 10^−4^) resulting in different distributions of transplant CMV risk with increased low risk in the ATG group (15.5 vs. 8%, *p* = 0.008) (Table 1).

### Impact of ATG on engraftment and GVHD

Engraftment and incidences of acute and chronic GVHD are shown in Table 2. There was no difference in terms of engraftment between the no-ATG and ATG groups (98.6 and 100%, respectively, *p* = 0.15). Median time for absolute nuclear cells (ANC) > 0.5 × 10^9^/L was longer in the no-ATG group (15 and 14 days, respectively, *p* = 0.001) (Table 2).

**Table 2:** Engraftment and GVHD

	No-ATG	ATG	*p* value
Total number of patients	421	145	
Engraftment, *n* (%)	411 (98.6%)	145 (100%)	0.15
No engraftment, *n* (%)	6 (1.4%)	0 (0%)	
Missing, *n*	5	0	
Median time ANC > 0.5 G/L (days, range)	15 (5–45)	14 (5–28)	0.001
Acute GVHD,			
Grade 0–I, *n* (%)	315 (78.2%)	116 (84.7%)	0.10
Grade II–IV, *n* (%)	88 (21.8%)	21 (15.3%)	
Grade III–IV, *n* (%)	31 (7.7%)	6 (4.4%)	0.19
Missing, *n*	5	3	
Chronic GVHD^a^			
All grades	52% (46–57.7)	30.8% (22.3–39.8)	0.00026
Extensive	26.3% (21.2–31.6)	7.6% (3.5–13.7)	4.7 × 10^−5^
Limited, *n*	71	26	
Extensive; *n*	77	8	
Missing, *n*	117	28	

*ATG* anti-thymocyte globulin, *GVHD* graft-versus-host disease

^a^Two-year cumulative incidence

**Fig. 1:**
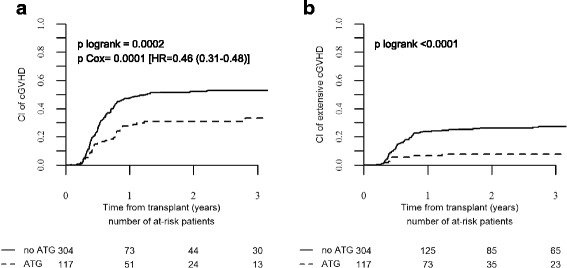
Cumulative incidence of chronic GVHD according to the use of ATG. **a** Overall incidence of chronic GVHD and **b** incidence of extensive chronic GVHD in the ATG and no-ATG groups as mentioned

In univariate analysis, we did not observe any impact of the use of ATG on the incidences of grade II–IV and grade III–IV aGVHD. Day 100 cumulative incidences of grade II–IV and III–IV aGVHD were similar between the no-ATG and ATG groups (21.8 vs. 15.3%, *p* = 0.10 and 7.7 vs. 4.4%, *p* = 0.19, respectively) (Table 2). By contrast, 2-year incidences of overall and extensive chronic GVHD were significantly reduced in the ATG group in comparison to the no-ATG group (30.8 vs. 52% for overall cGVHD, *p* = 0.0002 and 7.6 vs. 26.3% for extensive cGVHD, *p* < 10^−4^) (Tables 2 and 3 and Fig. 1). As shown in Table 4, GVHD-related deaths represented 22.2% (*n* = 32) and 17.1% (*n* = 6) of all causes of death in the no-ATG and ATG groups, respectively.

**Table 3:** Post-transplant 2-year outcomes

	NRM	RI	Extensive GVHD	GRFS	LFS	OS
No-ATG	17.3% [13.3–21.7]	27.2% [22.4–32.1]	26.3% [21.2–31.6]	39.6% [34–45.1]	55.4% [49.8–61]	58.9% [53.2–64.6]
ATG	10.7% [7.7–14.2]	22.5% [15.1–30.8]	7.6% [3.5–13.7]	60.1% [51–69.3]	66.8% [58.1–75.6]	71.8% [63.4–80.2]
*p* value	0.149	0.226	4.7 × 10^−5^	0.00016	0.044	0.049

*ATG* anti-thymocyte globulin, *GRFS* GVHD and relapse-free survival, *LFS* leukemia-free survival, *NRM* non-relapse mortality, *OS* overall survival, *RI* relapse incidence

**Table 4:** Causes of death

	No-ATG	ATG
	*N* = 144	*N* = 35
Relapse	77 (53.5%)	19 (54.3%)
GVHD	32 (22.2%)	6 (17.1%)
Infection	23 (16%)	5 (14.3%)
VOD	5 (3.5%)	3 (8.6%)
Idiopathic pneumonia	2 (1.4%)	0 (0%)
Hemorrhage	2 (1.4%)	0 (0%)
Other SCT-related	3 (2.1%)	2 (5.7%)

*ATG* anti-thymocyte globulin, *GVHD* graft-versus-host disease, *SCT* stem cell transplantation, *VOD* veno-occlusive disease

In multivariate analyses, the use of ATG was associated with a reduced risk of chronic GVHD development (hazard ratio (HR) = 0.46, 95% CI, 0.31–0.68; *p* = 0.0001) (Table 5). Factors associated with an increased risk of developing chronic GVHD were secondary AML (HR = 1.68, 95% CI, 1.04–2.72; *p* = 0.033) and the use of a female donor for a male recipient (HR = 1.75, 95% CI, 1.27–2.43; *p* = 0.001). We observed a center effect on the incidence cGVHD (*p* = 0.0007) (Table 5).

**Table 5:** Multivariate analyses

	Chronic GVHD		NRM		Relapse		LFS		OS		GRFS	
	*p* value	OR (95% CI)	*p* value	HR (95% CI)	*p* value	HR (95% CI)	*p* value	HR (95% CI)	*p* value	HR (95% CI)	*p* value	HR (95% CI)
In vivo T cell depletion	0.0001	0.46 (0.31–0.68)	0.094	0.59 (0.32–1.09)	0.149	0.72 (0.46–1.12)	0.027	0.67 (0.46–0.95)	0.027	0.65 (0.44–0.95)	4 × 10^−5^	0.51 (0.37–0.70)
Age at SCT >50 years	0.447	1.13 (0.82–1.57)	0.012	1.83 (1.14–2.94)	0.083	1.39 (0.96–2.00)	0.004	1.53 (1.15–2.05)	0.026	1.42 (1.04–1.93)	0.037	1.32 (1.02–1.71)
Interval diag. to SCT^a^	0.465	1.05 (0.91–1.22)	0.209	1.00 (1.00–1.00)	0.974	1.00 (1.00–1.00)	0.461	1.00 (1.00–1.00)	0.410	1.00 (1.00–1.00)	0.717	1.00 (1.00–1.00)
Year of SCT	0.929	1.00 (1.00–1.00)	0.894	0.99 (0.87–1.30)	0.862	1.01 (0.91–1.12)	0.960	1.00 (0.92–1.09)	0.558	0.98 (0.90–1.06)	0.522	0.98 (0.91–1.05)
Secondary AML	0.033	1.68 (1.04–2.72)	0.232	1.55 (0.76–3.19)	0.434	1.26 (0.71–2.26)	0.192	1.35 (0.86–2.12)	0.163	1.40 (0.87–2.25)	0.058	1.47 (0.99–2.18)
Female D to male R	0.001	1.75 (1.27–2.43)	0.669	1.12 (0.66–1.90)	0.319	1.22 (0.82–1.82)	0.306	1.18 (0.86–1.62)	0.253	1.21 (0.87–1.69)	0.0004	1.62 (1.24–2.11)
R CMV seropositivity	0.231	1.32 (0.84–2.09)	0.851	0.93 (0.46–1.90)	0.733	0.91 (0.55–1.53)	0.651	0.91 (0.60–1.38)	0.456	0.85 (0.55–1.31)	0.639	0.92 (0.64–1.32)
D CMV seropositivity	0.417	1.17 (0.81–1.68)	0.241	1.49 (0.77–2.89)	0.812	1.06 (0.67–1.68)	0.358	1. 20 (0.82–1.75)	0.359	1.21 (0.81–1.80)	0.832	1.04 (0.75–1.42)
Center (frailty variable)	0.0007		0.047		0.211		0.917		0.913		0.154	

*AML* acute myeloid leukemia, *CMV* cytomegalovirus, *D* donor, *diag* diagnosis, *GRFS* GVHD/relapse-free survival, *GVHD* graft-versus-host disease, *LFS* leukemia-free survival, *NRM* non-relapse mortality, *OS* overall survival, *R* recipient, *SCT* allogeneic stem cell transplantation

^a^Analyzed per 6-month interval

### Toxicity and NRM

**Fig. 2:**
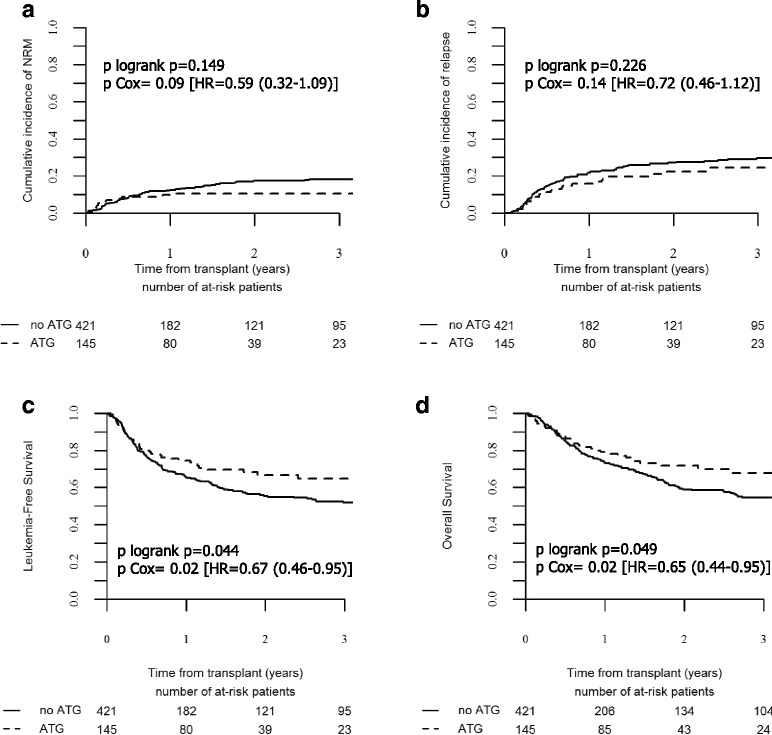
Transplant outcomes according to the use of ATG. Cumulative incidence of non-relapse mortality (NRM) (**a**), of relapse (**b**), leukemia-free survival (**c**), and overall survival (**d**) in the ATG and no-ATG groups as mentioned

The median follow-up of the entire cohort of 19 months (range, 1–106) was similar in both no-ATG (16 months) and ATG groups (21 months) (Table 1). Two-year NRM for the entire cohort was 15.5% (95% CI, 12.3–19.1). In univariate analysis, 2-year cumulative incidence of NRM was no different between the no-ATG (17.3%; 95% CI, 13.3–21.7) and the ATG (10.7%; 95% CI, 7.7–14.2) groups (*p* = 0.149). (Table 3 and Fig. 2a).

Apart from GVHD, the main other causes of death from NRM in the no-ATG and ATG groups were infections (23 patients and 5 patients (16 and 14.3% of all deaths), respectively) and veno-occlusive disease (3 patients in each group (3.5 and 8.6% of all deaths), respectively) (Table 4).

In multivariate analyses, recipient age above 50 years was associated with an increased risk of NRM (HR = 1.83, 95% CI, 1.14–2.94; *p* = 0.012) and we observed a center effect on the incidence of NRM (*p* = 0.047). Although not significant, there was a trend for reduced NRM in patients receiving ATG (HR = 0.59, 95% CI, 0.32–1.09; *p* = 0.094) (Table 5).

### Use of ATG had no impact on relapse incidence

Two-year cumulative incidence of relapse in the entire cohort of patients was 25.9% (95% CI, 21.8–30.1) and represented the main cause of death in the two groups of patients: 53.4% of all causes of death in the no-ATG and 54.5% of those of the ATG groups (Table 4). In univariate analysis, the use of ATG had no impact on the 2-year incidence of relapse, which occurred in 27.2% (95% CI, 22.4–32.1) of the patients in the no-ATG group and in 22.5% (95% CI, 15.1–30.8) of those in the ATG group (*p* = 0.226) (Table 3 and Fig. 2b). The absence of the impact of ATG on relapse risk was confirmed in multivariate analyses (HR = 0.72, 95% CI, 0.46–1.12; *p* = 0.149) (Table 5). No significant factor was associated with the risk of relapse in this study, although older age (>50 years) showed a trend to an increased risk (HR = 1.39, 95% CI, 0.96–2.00; *p* = 0.083).

### Use of ATG improved transplant survivals including GRFS

At 2 years, LFS and OS for in the entire cohort of patients were 58.4% (95% CI, 53.7–63.2) and 62.2% (95% CI, 57.4–67), respectively. In univariate analysis, 2-year LFS and OS were improved in the ATG group (66.8%; 95% CI, 58.1–75.6 and 71.8%; 95% CI, 63.4–80.2, respectively) in comparison to the no-ATG group (55.4%; 95% CI, 49.8–61 and 58.9%; 95% CI, 53.2–64.6, respectively) (*p* = 0.044 and 0.049, respectively) (Table 3 and Fig. 2c, d). The beneficial impact of ATG on LFS and OS was confirmed in multivariate analyses (HR = 0.67, 95% CI, 0.46–0.95, *p* = 0.027 for LFS and HR = 0.65; 95% CI, 0.44–0.95, *p* = 0.027 for OS) (Table 5). The only other factor that also impacted transplant survivals was recipient older age (>50 years) resulting in impaired LFS (HR = 1.53, 95% CI, 1.15–2.05, *p* = 0.004) and worse OS (HR = 1.42, 95% CI, 1.04–1.93, *p* = 0.026) (Table 5).

**Fig. 3:**
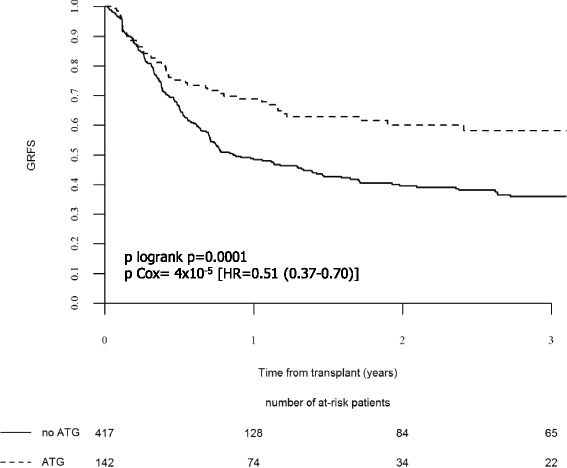
GRFS according to the use of ATG

Two-year overall GRFS was 45.0% (95% CI, 40.1–49.8). In univariate analysis, patients of the ATG group had improved 2-year GRFS (60.1%, 95% CI, 51–69.3) compared those of the no-ATG group (39.6%, 95% CI, 34–45.1) (*p* = 0.00016) (Table 3 and Fig. 3). Use of ATG was significantly associated with improved GRFS in multivariate analyses (HR = 0.51, 95% CI, 0.37–0.70, *p* = 4 × 10^−5^) (Table 5), while use of a female donor for a male recipient and recipient older age (>50 years) were associated with worse GRFS (HR = 1.62, 95% CI, 1.24–2.11, *p* = 0.0004 and HR = 1.32, 95% CI, 1.02–1.71, *p* = 0.037) (Table 5).

## Discussion

The main challenge of allo-SCT in AML and other hematological malignancies remains to limit organ life-threatening toxicity while preserving the GVL effect and patients’ quality of life by avoiding severe chronic GVHD. The GVHD and relapse-free survival composite end point is becoming an important end point to improve in allo-SCT [21, 23]. In this objective, while improvements in terms of tolerability of the transplant process have been shown in the last two decades by the development of reduced intensity and toxicity conditioning regimens and by improvement of supportive care including management of infections [24], the increased use of PBSC grafts, reaching 70% of stem cell grafts used in Europe nowadays [25], is also associated with higher incidence of severe cGVHD even with HLA-identical sibling donors [11, 12, 26], thus potentially impairing GRFS. The Flu-ivBu4 RTC associated with PBSC graft has been reported as effective than conventional Bu-cyclophosphamide MAC regimen but with reduced short- and long-term non-relapse mortality in AML patients transplanted in CR1 with an HLA 10/10-matched related or unrelated donor [8–10]. However, the incidence of chronic GVHD with PBSC (80%) grafts from HLA-identical sibling donors, following such RTC in the absence of ATG, remains high with 68% overall cGVHD and 42% extensive cGVHD incidences at 4 years post-transplant reported recently by the Spanish Cooperative Transplant Group [7]. The current study was limited to AML in CR1 transplanted with HLA-identical donors to reduce bias due to donor type and disease status on transplant outcomes. As reported in other contexts of allo-SCT [14, 16, 17, 27–30], we confirm in the present study that the addition of intermediate dose of thymoglobulin (median, 5 mg/kg) significantly reduces, after adjustment to other factors, the risk of developing cGVHD (Cox HR = 0.46, *p* = 0.0001). Compared to patients not receiving ATG, those transplanted with ATG, despite having received more frequently PBSC, had a reduction of 2 years of cumulative incidence of the overall cGVHD from 52 to 31% (*p* = 0.00026) and that of extensive cGVHD from 26 to 8% (*p* < 10^−4^). Such reduction of cGVHD incidence was not associated with reduced anti-leukemic control since use of ATG did not impact relapse incidence in this series of AML transplanted in CR1 in both univariate and multivariate analyses. Most patients had received a thymoglobulin dose of <6 mg/kg, so we could not analyze the impact of the ATG dose on outcomes. However, these results are in line with preserved GVL effect despite the addition of low or intermediate doses of ATG in the context of allo-SCT for AML performed with MRD and MUD following conventional MAC [31, 32] and RIC [29, 30, 33, 34], in contrast with increased risk of relapse with doses of thymoglobulin >10 mg/kg [28].

By contrast, as reported by others [15, 17, 30], we did not observe protective effect of such doses of ATG against acute GVHD. Actually, in the context of allo-SCT performed with PBSC from matched related or unrelated donors, use of low doses of ATG (2.5 mg/kg of thymoglobulin) was associated with an increased risk of aGVHD [34, 35]; a reduction of the incidence of aGVHD has been observed with thymoglobulin doses starting at 5 mg/kg, although higher doses (≥7.5 mg/kg of thymoglobulin) were associated with increased risk of mortality from infections and relapse in both MAC and RIC settings [28, 36]. The optimal dose of thymoglobulin in both RIC and MAC seems therefore to be about 5 mg/kg. At such intermediate dose of ATG, as described by others [14, 15, 30, 37], we did not observe difference of infection-related mortality between the ATG and no-ATG groups, despite potential increased frequency of viral EBV reactivation manageable by viral load monitoring and preemptive use of rituximab [30, 37].

Altogether, our results show that the addition of intermediate dose of ATG to the Flu-ivBu4 RTC represents an independent factor associated with improved GRFS, as defined by Ruggeri et al. for registry-based studies [21], allowing a probability of being alive without disease and without significant cGVHD at 2 years after allo-SCT in 60% of the patients transplanted for AML in CR1 with a sibling donor, compared to 40% of those not receiving ATG. Although GRFS is not routinely analyzed up to now, a 2-year 60% GRFS compares favorably to 40% GRFS at 3 years reported after allo-SCT performed for AML transplanted in CR1 (79%) or CR2 (21%) with MAC (61%) or RIC and PBSC (82%) from HLA-matched related (55%) or unrelated (45%) donors within the EBMT registry [21], and to 25% at 1 year reported with PBSC HLA-sibling donor allo-SCT by the Minnesota Group [23].

Use of ATG in our series appears also as an independent factor associated with improved LFS and OS, mainly due to reduced incidence of overall and extensive cGVHD leading to a trend towards increased late NRM in the absence of ATG. Although a center effect was observed in the incidence of cGVHD and NRM, possibly due to preferential used of ATG and of prophylactic donor lymphocyte infusion in some centers, we did not detect a center effect on LFS, OS, and GRFS. The only other factor associated with worse survivals was recipient age above 50 years, due to higher NRM, as reported by others with such conditioning regimen [7].

## Conclusions

We recognize that this study has several limitations, mainly because of its retrospective aspect and that the reason for the choice of GVHD prophylaxis was not known but mainly dependent on the center’s protocols. However, the study was performed on a homogeneous cohort of AML patients transplanted in CR1 with HLA-identical sibling donors following a Flu-ivBu4 RTC. Despite these limitations, the results of this study suggest that, in this particular setting, an intermediate dose of ATG improves the composite end point severe GVHD and relapse-free survival by reducing the incidence of overall and chronic GVHD without affecting the long-term anti-leukemic effect. These results should be confirmed in a well-designed phase III randomized trial.

## Additional file

Additional file 1: Table S1. List of institutions reporting the patients’ data for the study. (DOCX 33 kb)

## Abbreviations

aGVHD: Acute graft-versus-host disease
allo-SCT: Allogeneic hematopoietic stem cell transplantation
AML: Acute myeloid leukemia
ANC: Absolute nuclear cells
ATG: Anti-thymocyte globulin
BM: Bone marrow
cGVHD: Chronic graft-versus-host disease
CI: Cumulative incidence
CMV: Cytomegalovirus
CR: Complete remission
EBV: Epstein-Barr virus
Flu-ivBu4: Fludarabine and 4 days intravenous busulfan
GRFS: GVHD and relapse-free survival
GVL: Graft-versus-leukemia
HR: Hazard ratio
LFS: Leukemia-free survival
MAC: Myeloablative conditioning
MRD: Matched related donor
MUD: Matched unrelated donor
NRM: Non-relapse mortality
OS: Overall survival
PB: Peripheral blood
PBSC: Peripheral blood stem cells
RI: Relapse incidence
RTC: Reduced toxicity conditioning
VOD: Veno-occlusive disease
