# Supplementary material for: Impact of in vivo T cell depletion in HLA-identical allogeneic stem cell transplantation for acute myeloid leukemia in first complete remission conditioned with a fludarabine iv-busulfan myeloablative regimen: a report from the EBMT Acute Leukemia Working Party
Source: J Hematol Oncol. 2017 Jan 24;10:31. doi: 10.1186/s13045-016-0389-4 (PMC5259921; doi:10.1186/s13045-016-0389-4)
Supplement: Additional file 1: Table S1. — List of institutions reporting the patients’ data for the study. (DOCX 33 kb) [file 13045_2016_389_MOESM1_ESM.docx]

**Additional file 1: Table S1: List of institutions reporting patients’ data for the study**
